# 2156. The Rational Design of SER-147: a Cultivated Microbial Consortium to Restore Colonization Resistance and Mitigate Infection in Chronic Liver Disease

**DOI:** 10.1093/ofid/ofad500.1779

**Published:** 2023-11-27

**Authors:** Jenna Wurster, Tim Straub, Edward J O’Brien, Nathaniel J Ennis, Melissa Mayol, Jessica Brown, Marin Vulić, Nicholas Beauchemin, Elizabeth Halvorsen, Christopher Ford, Matthew Henn

**Affiliations:** Seres Therapeutics, Cambridge, Massachusetts; Seres Therapeutics, Cambridge, MA, Cambridge, Massachusetts; Seres Therapeutics, Cambridge, Massachusetts; Seres Therapeutics, Cambridge, Massachusetts; Seres Therapeutics, Cambridge, Massachusetts; Seres Therapeutics, Cambridge, Massachusetts; Seres Therapeutics, Cambridge, Massachusetts; Seres Therapeutics, Cambridge, Massachusetts; Seres Therapeutics, Cambridge, Massachusetts; Seres Therapeutics, Inc, Cambridge, MA; Seres Therapeutics, Cambridge, MA, Cambridge, Massachusetts

## Abstract

**Background:**

Chronic liver disease (CLD) is a major cause of morbidity and mortality, with high risk of infection as CLD progresses. Gut microbiome disruption, which worsens with CLD severity, causes loss of colonization resistance and impaired gastrointestinal (GI) barrier function, enabling pathogen translocation across the GI tract. We evaluated shotgun metagenomic data from 3 studies and 288 patients with CLD to evaluate the role of the microbiome in the progression of disease, and enable the rational design of SER-147, a cultivated consortium of human commensal bacteria, to restore colonization resistance against key pathogens and promote GI homeostasis in CLD patients.

**Methods:**

SER-147 design relied on both (1) microbiome targets identified from a bioinformatic analysis of public observational datasets in CLD patients and (2) past Seres clinical trial pharmacology data. We ingested genomic and matching clinical data from 3 studies and 288 patients (Table 1), processing all primary read data through Seres genomic databases to enable comparison with internal healthy comparator cohorts. Functional characterization of the Seres strain library (SSL) facilitated SER-147 design to include traits important to colonization resistance and GI barrier integrity (Figure 1).

**Table 1:**
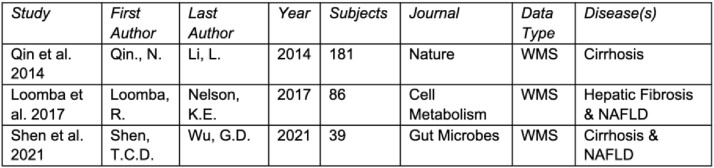
Public observational studies of patients with CLD used to evaluate the role of the microbiome in CLD progression.

**Figure 1.**
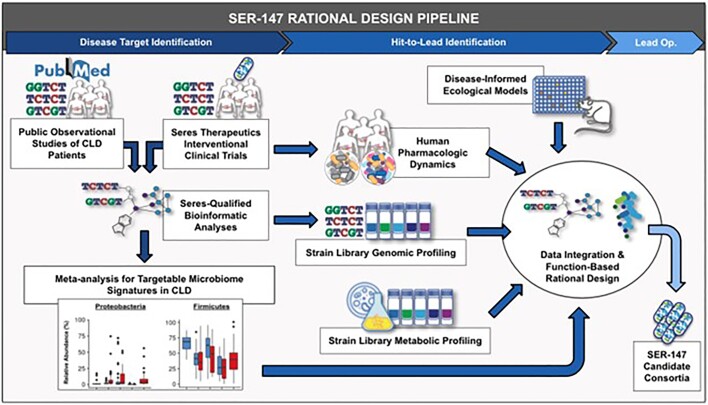
Reverse Translational Design Pipeline for SER-147 Candidate Consortia.

The design framework for preclinical drug candidates integrates a combination of public observational datasets in CLD patients, Seres interventional clinical trials, and proprietary bioinformatic algorithms to inform disease target identification. These data are subsequently fed into a rational design framework that utilizes (1) trial engraftment, (2) disease-informed preclinical microbiome modeling, and (3) functional learnings from SSL to identify taxa with the desired pharmacological properties.

**Results:**

Bioinformatic analyses support evidence of progressive microbiome disruption and diversity loss as CLD worsens, as key Firmicutes are reduced while Proteobacteria are enriched. Rational design yielded candidate consortia with taxa that can (1) generate barrier-restorative metabolites (short-chain fatty acids, secondary bile acids, tryptophan metabolites), (2) reduce inflammatory cytokine production, (3) tolerate oxidative and acid stress, and (4) decolonize intestinal pathogens in preclinical models (Figure 2).

**Figure 2.**
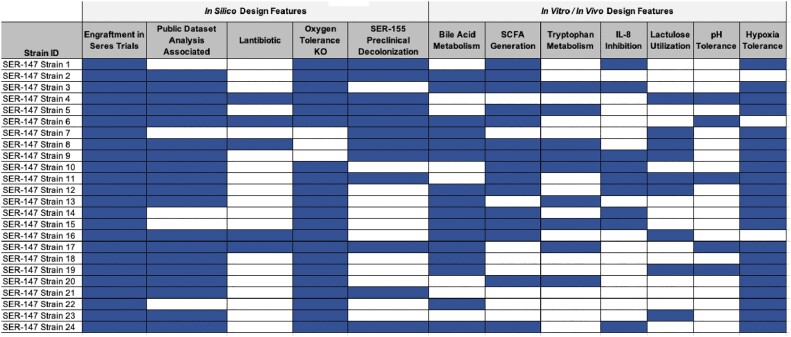
Selection of strains by baseline characteristics for use in SER-147.

Nineteen candidate bacterial strains were selected for inclusion in SER-147 candidate consortia based on numerous genotypic and phenotypic traits. Evidence of engraftment of strains in Seres clinical trials was a minimum inclusion criterion to help ensure pharmacokinetic capability of SER-147. Genomic and in silico design features included: depletion during CLD progression, predicted lantibiotic production, presence of KEGG-Orthologs for oxygen tolerance, and predicted contribution to decolonization phenotypes based on preclinical modeling. Phenotypic design features were selected based on extensive in vitro and in vivo characterization of strains and include the generation of barrier-restorative metabolites like short-chain fatty acids (SCFA), secondary bile acid conversion, and tryptophan metabolism. SER-147 candidate strains were additionally assessed in vitro for their capacity to tolerate low pH, grow in hypoxic culture conditions, suppress IL-8 activity, and utilize lactulose as a sole carbon source as it is a commonly prescribed medication in the CLD patient population

**Conclusion:**

Patients with CLD are at increased risk of mortality from infections seeded from the GI tract due to microbiome disruption and GI barrier compromise. Microbiome therapeutics such as SER-147 represent a potentially powerful and novel approach to reducing the risk of infection in CLD by directly targeting key risk factors of GI-seeded infections.

**Disclosures:**

**Jenna Wurster, PhD**, Seres Therapeutics: Paid Employee|Seres Therapeutics: Stocks/Bonds **Tim Straub, MS**, Seres Therapeutics: Employee|Seres Therapeutics: Stocks/Bonds **Edward J. O'Brien, PhD**, Seres Therapeutics: Employee|Seres Therapeutics: Stocks/Bonds **Nathaniel J. Ennis, n/a**, Seres Therapeutics: Employee|Seres Therapeutics: Stocks/Bonds **Melissa Mayol, n/a**, Seres Therapeutics: Employee|Seres Therapeutics: Stocks/Bonds **Jessica Brown, n/a**, Seres Therapeutics: Employee|Seres Therapeutics: Stocks/Bonds **Marin Vulić, PhD**, Seres Therapeutics: Employee|Seres Therapeutics: Stocks/Bonds **Nicholas Beauchemin, n/a**, Seres Therapeutics: Employee|Seres Therapeutics: Stocks/Bonds **Elizabeth Halvorsen, PhD**, Seres Therapeutics: Employee|Seres Therapeutics: Stocks/Bonds **Christopher Ford, PhD**, Seres Therapeutics: Employee|Seres Therapeutics: Stocks/Bonds **Matthew Henn, PhD**, Seres Therapeutics: Employee|Seres Therapeutics: Stocks/Bonds

